# Pembrolizumab combined with disitamab vedotin in human epidermal growth factor receptor 2-positive metastatic colorectal cancer

**DOI:** 10.1093/oncolo/oyag385

**Published:** 2026-10-01

**Authors:** Xiaojing Xu, Minzhi Lv, Luoyan Ai, Qing Wang, Yuehong Cui, Wei Li, Qing Liu, Xi Tang, Xi Chen, Shan Yu, Mengzhou Guo, Yiyi Yu, Tianshu Liu

**Affiliations:** Department of Oncology, Zhongshan Hospital, Fudan University, Shanghai, 200032, China; Cancer Center, Zhongshan Hospital, Fudan University, Shanghai, 200032, China; Cancer Center, Zhongshan Hospital, Fudan University, Shanghai, 200032, China; Department of Cancer Screening and Prevention, Zhongshan Hospital, Fudan University, Shanghai, 200032, China; Evidence-based Medicine Center, Fudan University, Shanghai, 200032, China; Department of Oncology, Zhongshan Hospital, Fudan University, Shanghai, 200032, China; Cancer Center, Zhongshan Hospital, Fudan University, Shanghai, 200032, China; Department of Oncology, Zhongshan Hospital, Fudan University, Shanghai, 200032, China; Cancer Center, Zhongshan Hospital, Fudan University, Shanghai, 200032, China; Department of Oncology, Zhongshan Hospital, Fudan University, Shanghai, 200032, China; Cancer Center, Zhongshan Hospital, Fudan University, Shanghai, 200032, China; Department of Oncology, Zhongshan Hospital, Fudan University, Shanghai, 200032, China; Cancer Center, Zhongshan Hospital, Fudan University, Shanghai, 200032, China; Department of Oncology, Zhongshan Hospital, Fudan University, Shanghai, 200032, China; Cancer Center, Zhongshan Hospital, Fudan University, Shanghai, 200032, China; Department of Oncology, Huadong Hospital, Fudan University, Shanghai, 200040, China; Department of Oncology, Huadong Hospital, Fudan University, Shanghai, 200040, China; Department of Oncology, Zhongshan Hospital, Fudan University, Shanghai, 200032, China; Cancer Center, Zhongshan Hospital, Fudan University, Shanghai, 200032, China; Department of Oncology, Zhongshan Hospital, Fudan University, Shanghai, 200032, China; Cancer Center, Zhongshan Hospital, Fudan University, Shanghai, 200032, China; Department of Oncology, Zhongshan Hospital, Fudan University, Shanghai, 200032, China; Cancer Center, Zhongshan Hospital, Fudan University, Shanghai, 200032, China; Department of Oncology, Zhongshan Hospital, Fudan University, Shanghai, 200032, China; Cancer Center, Zhongshan Hospital, Fudan University, Shanghai, 200032, China; Evidence-based Medicine Center, Fudan University, Shanghai, 200032, China

**Keywords:** pembrolizumab, disitamab vedotin, HER2, metastatic colorectal cancer

## Abstract

**Background:**

Late-line treatment options for microsatellite stable (MSS)/proficient mismatch repair (pMMR) metastatic colorectal cancer (mCRC) are limited. PD-1 blockade alone has minimal clinical benefit in this population. Human epidermal growth factor receptor 2 (HER2)-targeted therapies represent an emerging strategy for HER2-expressing mCRC. This study evaluated the efficacy and safety of pembrolizumab combined with disitamab vedotin (DV), an anti-HER2 antibody–drug conjugate (ADC), in pretreated patients with HER2-positive mCRC.

**Methods:**

This multicenter, single-arm, phase II study enrolled patients with MSS/pMMR, HER2-positive mCRC who received 2 prior lines of systemic therapy. Patients received pembrolizumab 400 mg every 6 weeks plus DV 2.5 mg/kg every 2 weeks until disease progression, unacceptable toxicity, or death. The primary endpoint was objective response rate (ORR). Secondary endpoints included progression-free survival (PFS), overall survival (OS), and safety.

**Results:**

Between December 2022 and May 2024, 15 patients (median age 59.0 years; 60.0% male) were enrolled. Fourteen patients (93.3%) had HER2 IHC 3+ expressions, and 2 had received prior anti-HER2 therapy. Two patients achieved partial response (ORR = 13.3%; 95% CI, 1.7%-40.5%). With a median follow-up of 26.7 months, median PFS was 2.9 months (95% CI, 2.3 to not reached). Median OS was not reached (95% CI, 16.7 to NR). The 6-month OS rate was 92.9% (95% CI: 6.9%-80.3%), and the 1-year OS rate was 71.4% (95% CI: 51.3%-99.5%). The most common treatment-related adverse events (TRAEs) included nausea, anorexia, alanine aminotransferase elevation, aspartate aminotransferase elevation, and alopecia. Grade 3-4 TRAEs occurred in 2 patients (14.3%), both neutropenia.

**Conclusions:**

Pembrolizumab plus disitamab vedotin showed limited antitumor activity in pretreated HER2-positive mCRC.

**ClinicalTrials.gov Identifier:**

NCT05333809

Lessons learned

- Key outcome: The combination of pembrolizumab plus disitamab vedotin showed minimal antitumor activity in HER2-positive metastatic colorectal cancer (ORR 13.3%, median PFS 2.9 months), leading to early study termination.
- Key lesson: Microtubule inhibitor–based ADCs (MMAE payload) appear unlikely to be effective in colorectal cancer, even when combined with PD-1 blockade.
- Key reason: MMAE-based ADCs may be less effective in HER2-positive colorectal cancer, potentially due to MDR1-mediated drug efflux, weak reliance on microtubule dynamics, and limited bystander killing—although these mechanistic explanations remain speculative without correlative biomarker data.

## Trial information

**Table 1.** Efficacy outcomes in the intention-to-treat population.

PEMBROLIZUMAB AND DISTAMAB VEDOTIN (*N* = 15)	
**Complete response**	0
**Partial response**	2 (13.3)
**Stable disease**	6 (40.0)
**Disease progression**	6 (40.0)
**Not evaluable**	1 (6.7)
**Objective response, *n* (%)**	2(13.3)
**Objective response, % (95% CI)**	1.7-40.5
**Disease control, *n* (%)**	8 (53.3)
**Disease control, % (95% CI)**	26.6-78.7
**Median PFS (95% CI), months**	2.9 (2.3-NR)
**Median OS (95% CI), months**	NR (16.3 to NR)
**6-month OS rate, % (95% CI)**	92.9 (6.9-80.3)
**12-month OS rate, % (95% CI)**	71.4 (51.3-99.5)
**18-month OS rate, % (95% CI)**	64.3 (43.5-95.0)

**Table:** 

**TRIAL INFORMATION**	
**Disease**	Human epidermal growth factor receptor 2-positive metastatic colorectal cancer
**Stage of Disease/treatment**	IV
**Prior therapy**	At least 2 prior regimens
**Type of study**	Phase II
**Primary endpoints**	Objective response rate (ORR)
**Secondary endpoints**	Progression-free survival (PFS), overall survival (OS), disease control rate (DCR), safety, and tolerability
**Additional details of endpoints or study design: Patients must have histologically confirmed HER2-positive [According to the HERACLES criteria, HER2 positivity is defined as IHC 3 + (circumferential membrane staining in ≥50% of tumor cells, or in 10%-50% with FISH positivity) or IHC 2 + (moderate staining in ≥50% of tumor cells) with FISH amplification (HER2/CEP17 ratio ≥2.0)] colorectal adenocarcinoma that failed at least 2 prior lines of systemic therapy. Patients with mismatch repair-deficient, microsatellite instability-high, or RAS/BRAF mutations, prior immunotherapy or other HER2-ADC treatment, and active autoimmune diseases were excluded from this study.**	

## Drug information

**Table:** 

DRUG INFORMATION	
**Generic/working name**	Pembrolizumab; disitamab vedotin
**Company name**	Merck & Co., Inc.; Rongde Bio (RCABio)
**Drug type**	Immune checkpoint inhibitor; antibody–drug conjugate (ADC)
**Drug class**	Anti-PD-1 monoclonal antibody; anti-HER2 ADC with payload of monomethyl auristatin E (MMAE)
**Dose**	Pembrolizumab at a dose of 400 mg; disitamab vedotin at a dose of 2.5 mg/kg
**Route**	IV
**Schedule of administration**	Pembrolizumab at Days 1 of 6-week cycle; disitamab vedotin at Days 1 of 2-week cycle

## Patient characteristics

**Table:** 

PATIENT CHARACTERISTICS	
**Number of patients, male**	9
**Number of patients, female**	6
**Stage**	IV
**Median age (years)**	59
**Median number of systemic therapies**	2
**Number of prior systemic therapies: 2**	9
**Number of prior systemic therapies: 3**	6
**Performance status: ECOG 0 or 1**	15
**Performance status: ECOG 2 or above**	0
**HER2 expression (IHC 3+)**	14
**HER2 expression (IHC 2+ and FISH positive)**	1
**Median tumor burden in the target lesion ≥50 mm**	8
**Median tumor burden in the target lesion ≥100 mm**	3
**Primary tumor site: left side**	13
**Primary tumor site: right side**	2
**Number of metastatic organs: 1 or 2**	7
**Number of metastatic organs: 3 or above**	8
**Metastatic sites: liver**	12
**Metastatic sites: lung**	12
**Metastatic sites: lymph node**	5
**Prior therapy including oxaliplatin**	15
**Prior therapy including irinotecan**	15
**Prior therapy including cetuximab**	5
**Prior therapy including 5-FU**	12
**Prior therapy including bevacizumab**	14
**Prior therapy including capecitabine**	7
**Prior therapy including fruquintinib**	1
**Prior therapy including regorafenib**	1
**Prior therapy including pyrotinib**	2
**Prior therapy including trastuzumab**	1

## Primary assessment method

The study was designed using Simon’s 2-stage minimax design. A sample size of 25 patients was planned to demonstrate an improvement in ORR from 15% to 40%, with a one-sided type I error rate of 0.05 and power of 0.80. In the first stage, 7 patients were to be enrolled; if one or fewer responses were observed, the trial would be terminated for futility. If 2 or more responses were observed, enrollment would proceed to the second stage with an additional 18 patients (total 25). Accounting for a 10% follow-up loss rate, the total target sample size was 30 patients.

Between December 2022 and May 2024, 17 patients were screened, and 2 did not meet the eligibility criteria. Seven patients were enrolled in the first stage, and 2 partial responses (PR) were observed, meeting the prespecified criteria to proceed. An additional eight patients were enrolled in the second stage, but no further responses occurred. Given that the overall response rate (2/15, 13.3%) fell far below the anticipated 40%, and the conditional power for achieving the primary endpoint was negligible, the trial was terminated early for futility. Thus, a total of 15 patients were enrolled. Among them, one withdrew informed consent before treatment, leaving 14 patients who received at least one cycle of study treatment.

The primary endpoint was ORR, defined as the proportion of the subjects in the analysis population with the best response of complete response (CR) or PR. Patients whose disease was not assessed and those who were unavailable for the follow-up were considered as nonresponders for the primary endpoint analysis. Secondary endpoints were progression-free survival (PFS), overall survival (OS), disease control rate (DCR), safety and tolerability. PFS was defined as the duration from the treatment initiation to disease progression or death, whichever occurred first. OS was defined as the duration from the treatment initiation to death. DCR was defined as the proportion of patients achieving CR, PR, or stable disease (SD).

**Table:** 

PRIMARY ASSESSMENT METHOD	
**Title**	ORR, DCR, OS, PFS, safety
**Number of patients screened**	17
**Number of patients enrolled**	15
**Number of patients evaluable for toxicity**	14
**Number of patients evaluated for efficacy**	15
**Evaluation method**	Tumor was assessed by investigators using enhanced computed tomography or magnetic resonance imaging per the Response Evaluation of Criteria in Solid Tumor version 1.1 (RECIST v1.1) at baseline and every 6 weeks. Safety was assessed by monitoring adverse events (AEs) by the National Cancer Institute’s Common Toxicity Criteria for Adverse Events version 5.0.
**Outcome notes: efficacy outcomes (see Table 1, Figures 1 and 2).**	

**Figure 1.**
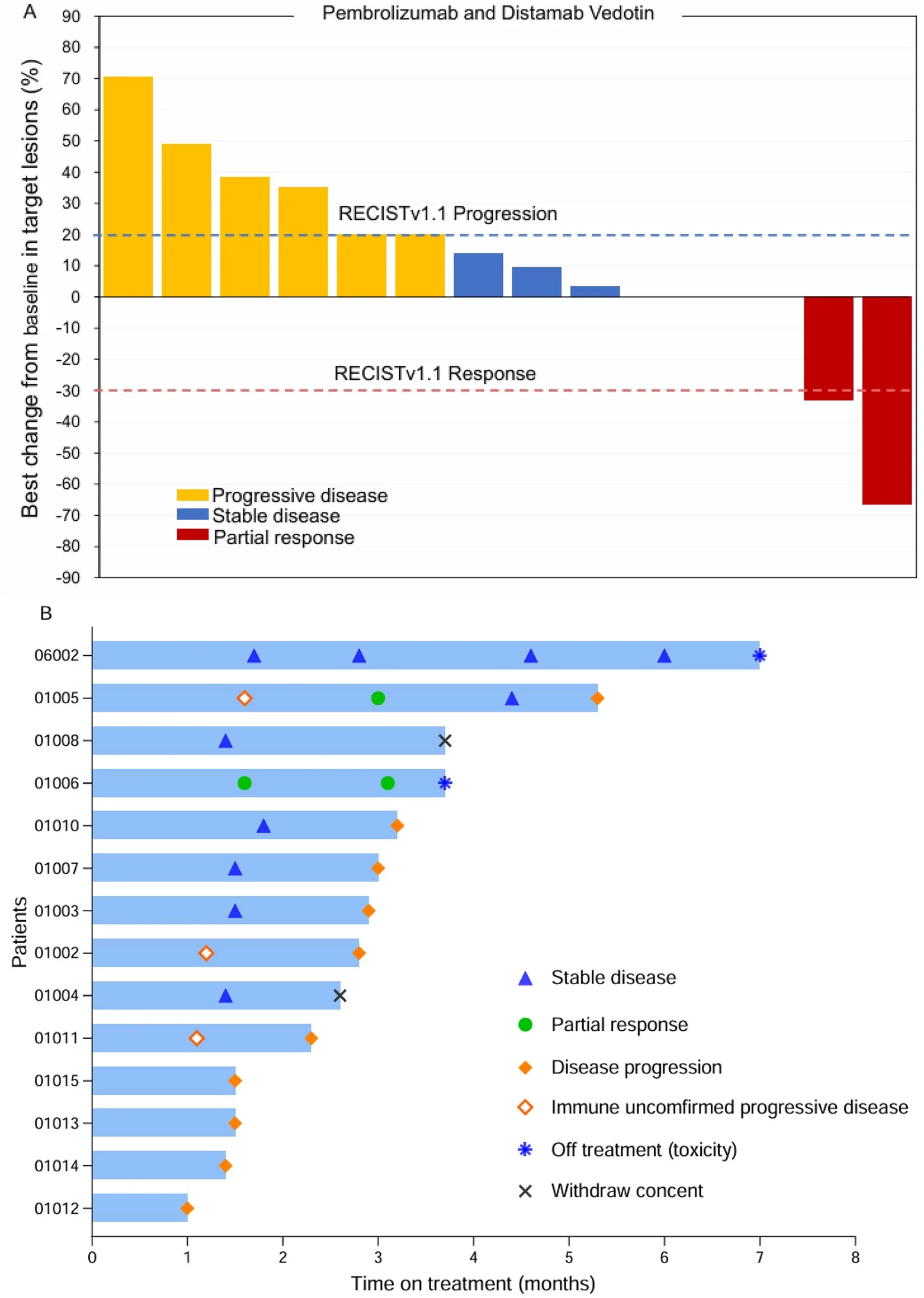
Response outcomes and study treatment. (A) Waterfall plot of tumor changes from baseline. (B) Swimmer plot representing the duration of treatment.

**Figure 2.**
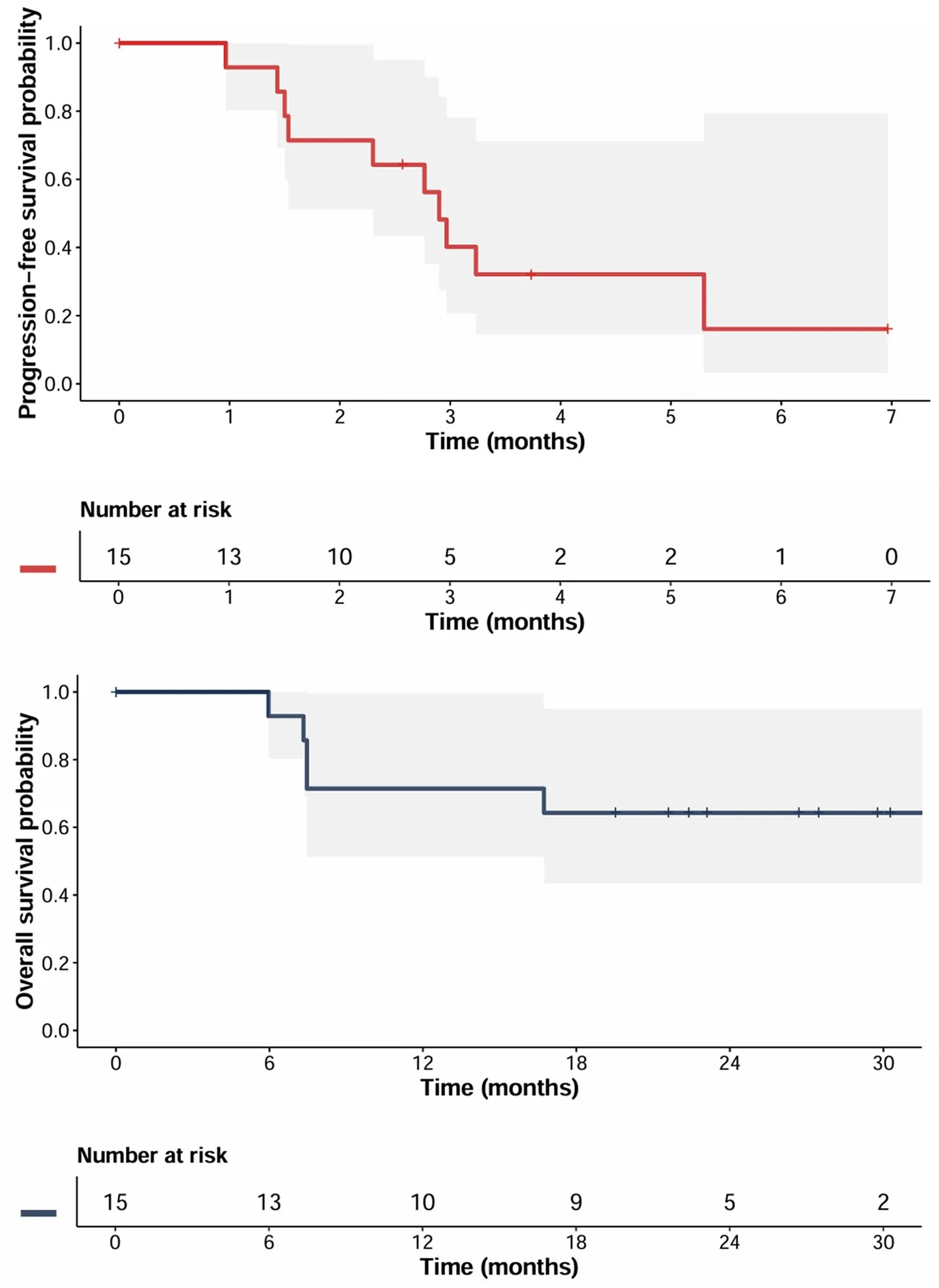
Survival outcomes in the intention-to-treat population. (A) Kaplan–Meier curves of PFS. (B) Kaplan–Meier curves of OS. Data cutoff date: December 29, 2025.

## Serious adverse events (SAEs)

No SAEs occurred in this study.

## General toxicity profile

Adverse events table using CTCAE:

**Table:** 

EVENTS	ANY GRADE	GRADE 3-4
**Nausea**	78.6% (11/14)	0% (0/14)
**Anorexia**	78.6% (11/14)	0% (0/14)
**Alanine aminotransferase increased**	78.6% (11/14)	0% (0/14)
**Aspartate aminotransferase increased**	78.6% (11/14)	0% (0/14)
**Alopecia**	71.4% (10/14)	0% (0/14)
**Numbness in limbs**	57.1% (8/14)	0% (0/14)
**Alkaline phosphatase increased**	50% (7/14)	0% (0/14)
**Fatigue**	50% (7/14)	0% (0/14)
**Neutrophil count decreased**	35.7% (5/14)	14.3% (2/14)
**Dermatitis**	35.7% (5/14)	0% (0/14)
**Vomiting**	28.6% (4/14)	0% (0/14)
**White cell count decreased**	21.4% (3/14)	7.1% (1/14)
**Abdominal pain**	21.4% (3/14)	0% (0/14)
**Constipation**	21.4% (3/14)	0% (0/14)
**Weight loss**	21.4% (3/14)	0% (0/14)
**Rash**	14.3% (2/14)	0% (0/14)
**Diarrhea**	14.3% (2/14)	0% (0/14)
**Anemia**	14.3% (2/14)	0% (0/14)
**Proteinuria**	14.3% (2/14)	0% (0/14)
**Platelet count decreased**	14.3% (2/14)	0% (0/14)
**Hypothyroidism**	14.3% (2/14)	0% (0/14)
**Fever**	14.3% (2/14)	0% (0/14)
**Cough**	14.3% (2/14)	0% (0/14)
**Hypertension**	14.3% (2/14)	0% (0/14)
**Hyperglycemia**	14.3% (2/14)	0% (0/14)
**Hypokalemia**	14.3% (2/14)	0% (0/14)
**Creatine phosphokinase increased**	7.1% (1/14)	0% (0/14)
**Hyperthyroidism**	7.1% (1/14)	0% (0/14)
**Night sweats**	7.1% (1/14)	0% (0/14)
**Dizziness**	7.1% (1/14)	0% (0/14)
**Limb pain**	7.1% (1/14)	0% (0/14)
**Cholecystitis**	7.1% (1/14)	0% (0/14)
**Blood bilirubin increased**	7.1% (1/14)	0% (0/14)
**Serum amylase increased**	7.1% (1/14)	0% (0/14)
**Palpitations**	7.1% (1/14)	0% (0/14)
**Chest tightness**	7.1% (1/14)	0% (0/14)
**Hypertriglyceridemia**	7.1% (1/14)	0% (0/14)
**Blood in stool**	7.1% (1/14)	0% (0/14)
**Cardiac troponin T increase**	7.1% (1/14)	0% (0/14)
**Creatinine increased**	7.1% (1/14)	0% (0/14)
**Sore fingers**	7.1% (1/14)	0% (0/14)

## Discussion

This phase II study evaluated pembrolizumab plus disitamab vedotin in 15 patients with pretreated HER2-positive mCRC. The combination demonstrated limited efficacy (ORR 13.3%, mPFS 2.9 months), leading to early termination. Despite these negative results, several clinically relevant insights emerge that may inform future therapeutic development.

First, this study reinforces the intrinsic resistance of pMMR CRC to immunotherapy. While ICIs demonstrate remarkable efficacy in dMMR CRC,^1^ pMMR tumors—characterized by low tumor mutational burden, low PD-L1 expression, and sparse tumor-infiltrating lymphocytes^2^—remain largely refractory to immunotherapy even when combined with targeted agents. Our findings add to the growing evidence that current combinatorial strategies in pMMR mCRC yield suboptimal or non-reproducible results,^3–5^ highlighting the need for more effective approaches to remodel the “immune-cold” microenvironment.

Second, ADC payload selection may be an important determinant of efficacy in CRC. The limited activity of MMAE payload in CRC^6–8^ could be related to several factors, including MDR1/ABCB-mediated drug efflux^9^^,^^10^; the possibility that CRC, driven largely by EGFR/RAS/RAF/PI3K signaling, may have relatively weak dependence on microtubule dynamics^6^^,^^8^^,^^11^; and the modest bystander effect in the dense CRC microenvironment.^12^^,^^13^ However, these mechanistic explanations remain speculative, as our study lacked correlative biomarker analyses. Notably, 46.7% of patients in our study subsequently received trastuzumab deruxtecan or SHR-A1811^14^^,^^15^ (another topoisomerase I inhibitor-loaded HER2-ADC) after discontinuing the study regimen and achieved prolonged disease control, with PFS exceeding 6 months and an 18-month OS rate above 60%. While limited by small numbers and potential selection bias, this observation is hypothesis-generating and suggests that topoisomerase I inhibitor-based ADCs may warrant further investigation in HER2-positive CRC, a hypothesis that requires validation in dedicated prospective studies.^16^

Third, the limited synergy between disitamab vedotin and pembrolizumab suggests the need for alternative combination strategies. MMAE-induced immunogenic cell death may be insufficient to prime meaningful T-cell responses in CRC. Moreover, concurrent administration may not be optimal; a sequential approach (ADC first for immune priming, then PD-1 blockade) may more effectively potentiate antitumor immunity. The KEYNOTE-811 paradigm in HER2-positive gastric cancer (trastuzumab plus pembrolizumab),^17^^,^^18^ merits evaluation in HER2-positive mCRC, as trastuzumab exerts pleiotropic effects including ADCC and may synergize more effectively with checkpoint inhibitors.^19^^,^^20^ These mechanistic explanations, however, remain speculative and should be interpreted with caution.

The safety profile of pembrolizumab plus disitamab vedotin was generally manageable and consistent with the known toxicities of both agents. All 14 evaluable patients experienced treatment-related adverse events, predominantly grade 1–2 gastrointestinal, hepatic, constitutional, and dermatological toxicities, alopecia, and peripheral neuropathy. Grade 3-4 events were limited to neutropenia (14.3%), and no treatment-related deaths occurred. Immune-related adverse events occurred in 35.7% of patients (all grades 1-2), most commonly thyroid dysfunction. Treatment discontinuation due to TRAEs was low (14.3%).

Several limitations of this study must be acknowledged. The small sample size precludes definitive conclusions and may underestimate or overestimate the true treatment effect. The absence of mandatory pretreatment biopsies and correlative ctDNA analyses precludes definitive mechanistic conclusions, and any proposed mechanisms remain speculative. Additionally, the heterogeneity of prior therapies, including prior anti-HER2 treatment in 2 patients, may have influenced outcomes. Despite these limitations, this study represents—to our knowledge—the first prospective evaluation of a PD-1 inhibitor combined with an anti-HER2 ADC specifically in HER2-positive mCRC.

In conclusion, this study failed to demonstrate meaningful efficacy of pembrolizumab combined with disitamab vedotin in HER2-positive mCRC. Future investigations should prioritize HER2-ADCs with topoisomerase I inhibitor payloads and explore alternative combinatorial approaches, including monoclonal antibody with immunotherapy, in carefully selected patient subsets.

## Data Availability

The datasets used and/or analyzed during the current study are available from the corresponding author upon reasonable request (Tianshu Liu, e-mail: liu.tianshu@zs-hospital.sh.cn).
